# The National Hospital for the Paralysed and Epileptic, Queen Square, Bloomsbury

**Published:** 1904-10-15

**Authors:** 


### THE NATIONAL HOSPITAL FOR THE PARALYSED AND EPILEPTIC, QUEEN SQUARE, BLOOMSBURY.

### OPENING OF THE NEW OPERATING THEATRE.

On Saturday in last week, H.R.H., the Duchess of Albany, opened a new operating theatre at the National Hospital for the Paralysed and Epileptic. In 1882 the late Duke of Albany publicly announced his intention of erecting the main building, and in due course the work was begun. But as the Duke died before the undertaking was completed, the western wing, which was opened in 1885, was dedicated to his memory.

The Westminster wing, accommodating the surgical department, was opened by the late Duke of Westminster in 1891, and the theatre was constructed to serve both as a lecture-room and an operating theatre, an expedient inconsistent with the modern requirements of surgery.

The hospital having recently received £500 from King Edward's Fund, £150 from the Drapers' Company, and other donations, the Board resolved, with these sums as the nucleus of a fund, to construct a new theatre.

For the opening ceremony the invited guests assembled in the Dettmar ward.

The Duchess of Albany was accompanied by her daughter, Princess Alexander of Teck. Among those present were Bishop Welldon, the Dean of Canterbury, Lady Gowers, Sir Victor and Lady Horsley, Sir Felix Semon, Lady Russell Reynolds, and Mrs. J. Danvers Power.

The Duchess was received by the chairman (Mr. John Danvers Power) who welcomed her and read her an address, in which was set forth that the hospital authorities were endeavouring to carry on work worthy of the late Duke's honoured name.

The Duchess replied, thanking him for his kind welcome and saying she could not but feel a great interest in the work of the hospital and that she would do all in her power to help it on. She thanked the board of management for doing all they could to perpetuate her husband's memory: she was also grateful to them for having borne in mind her suggestion respecting the operating theatre. Before formally declaring the building open she asked those present to join in the earnest hope that all the success which human care and human skill could command would follow the work undertaken within these walls. Her Royal Highness then declared the new theatre open.

Bishop Welldon offered prayer, and then Sir Victor Horsley, speaking on behalf of the medical staff, asked the Duchess to accept their thanks for inaugurating what was really a new era in the development of the surgical work of that hospital. That it had passed the elementary stage was marked by the opening of the new theatre with every modern appliance. He went on to speak of the many benefits that had been the outcome of Lord Lister's antiseptic surgery, and he thought that the new theatre might justly be dedicated to Lord Lister. He had pleasure in announcing that £1,000 had been received from the Jessie Alice Palmer Charitable Fund, subject to the discretion of Mr. Heron Allem, who devoted the sum to the endowment of a work-table in the museum.

After the ceremony, the Duchess of Albany inspected the wards and the new theatre; and later in the afternoon attended the harvest festival service held in the hospital chapel.

The new operating theatre, though unpretentious, seems completely efficient, and capable of meeting every demand which may be made upon it. The appliances, including the apparatus for lighting and heating, are of the best, and the work has been carried on under the supervision of Sir Victor Horsley and Mr. Ballance (the surgeons at the hospital), who are entirely satisfied with the result. The architect is Mr. R. Langton Cole, F.R.I.B.A., and the builders and contractors, Messrs. J. Simpson and Son. The total cost is £1,200. The size of the theatre, which is cornerless, is 14 feet by 20 feet. The walls are of white opalite tiles, relieved by one row of blue-green tiles all the way round. The floor is of marble terrazzo pavement, and there is a glazed roof. There are two large windows, the upper parts of which are made of ordinary glass, while the lower parts are glazed with cathedral glass to prevent observation; the frames are of gun-metal. The students' gallery, which has standing room for 20 students, consists of two long white marble steps and a white enamelled railing. The shelves to hold lotion bottles are made of newellite glass shelving on galvanised iron brackets. The new theatre basins are by Doulton; but the theatre furniture, by Mayer and Meltzer, is from the old theatre, retained on account of its being quite modern and efficient.

In the adjoining anæsthetic room the skylight has Helliwell's galvanised iron patent glazing bars with lead coverings, securely fixed to ridge and curb, without wood in any shape, and glazed with Pilkington's wired sheet glass. The small sterilising room has a similar skylight.

The theatre heating apparatus, which is quite new, is worked by steam, and the makers are Messrs. Rosser and Russell, Charing Cross. The heating coils are enclosed in white enamelled doors with gun-metal handles, to allow the regulation of the heat. These doors can be opened and the coils pulled out. Messrs. Sunderland and Co. have supplied the electric light fittings, which include six lights in the theatre, four of which, shaded with green glass, are over the operating table. There is a connection for a lamp on the surgeon's forehead, and also for a portable lamp. The electric ventilating fan is one of high speed by Sunderland and Co.

